# New In Vitro-In Silico Approach for the Prediction of In Vivo Performance of Drug Combinations

**DOI:** 10.3390/molecules26144257

**Published:** 2021-07-13

**Authors:** Cristiana Correia, Abigail Ferreira, Joana Santos, Rui Lapa, Marjo Yliperttula, Arto Urtti, Nuno Vale

**Affiliations:** 1 OncoPharma Research Group, Center for Health Technology and Services Research (CINTESIS), Rua Dr. Plácido da Costa, 4200-450 Porto, Portugal; cristianacorreia95@gmail.com (C.C.); abigail.ferreira@fc.up.pt (A.F.); jmdmsantos@hotmail.com (J.S.); 2 Faculty of Sciences, University of Porto, Rua do Campo Alegre, s/n, 4169-007 Porto, Portugal; 3 LAQV/REQUIMTE, Laboratory of Applied Chemistry, Department of Chemical Sciences, Faculty of Pharmacy, University of Porto, Rua de Jorge Viterbo Ferreira, 228, 4050-313 Porto, Portugal; ruilapa@ff.up.pt; 4 Division of Pharmaceutical Biosciences, Faculty of Pharmacy, University of Helsinki, P.O. Box 56, 00014 Helsinki, Finland; marjo.yliperttula@helsinki.fi (M.Y.); arto.urtti@helsinki.fi (A.U.); 5 MEDCIDS—Department of Community Medicine, Health Information and Decision, Faculty of Medicine, University of Porto, Alameda Prof. Hernâni Monteiro, 4200-319 Porto, Portugal

**Keywords:** in vitro-in silico approach, pharmacokinetics, drug repurposing, drug combination, cell growth inhibition

## Abstract

Pharmacokinetic (PK) studies improve the design of dosing regimens in preclinical and clinical settings. In complex diseases like cancer, single-agent approaches are often insufficient for an effective treatment, and drug combination therapies can be implemented. In this work, in silico PK models were developed based on in vitro assays results, with the goal of predicting the in vivo performance of drug combinations in the context of cancer therapy. Combinations of reference drugs for cancer treatment, gemcitabine and 5-fluorouracil (5-FU), and repurposed drugs itraconazole, verapamil or tacrine, were evaluated in vitro. Then, two-compartment PK models were developed based on the previous in vitro studies and on the PK profile reported in the literature for human patients. Considering the quantification parameter area under the dose-response-time curve (AUC_effect_) for the combinations effect, itraconazole was the most effective in combination with either reference anticancer drugs. In addition, cell growth inhibition was itraconazole-dose dependent and an increase in effect was predicted if itraconazole administration was continued (24-h dosing interval). This work demonstrates that in silico methods and AUC_effect_ are powerful tools to study relationships between tissue drug concentration and the percentage of cell growth inhibition over time.

## 1. Introduction

The process of research and development (R&D) of new drugs is very time consuming and expensive. New drugs approval takes on average seven to nine years and the cost of introducing a new drug can range from 600 million to 1 billion euros [1,2]. Pharmacokinetic (PK) models describe the absorption, distribution, metabolism, and the elimination of molecules (drugs, compounds under development, etc.) in an organism, thus providing useful information to foster efficient and informative drug development. These models not only improve decision making throughout clinical drug development, but also enable the design and optimization of dosing regimens, increasing the chances of the drug to reach its target with the desired concentration and drug plasma concentration to be maintained within the therapeutic window [3,4,5]. The study of PK involves both theoretical and experimental approaches. Theoretical approaches aim at the development of PK models to predict drug disposition, which includes drugs distribution and elimination after its administration. These models can be divided into three categories, empirically, physiologically, and compartmentally based PK models [6]. The most used PK model is the compartmentally based, which represents a very simple and useful tool in PK. In this model type, tissues are grouped into compartments, depending on their blood flow and drug binding (tissues with similar blood flow and drug tissue binding are grouped in the same compartment) [3,4,5,7,8,9].

**Figure 1:**
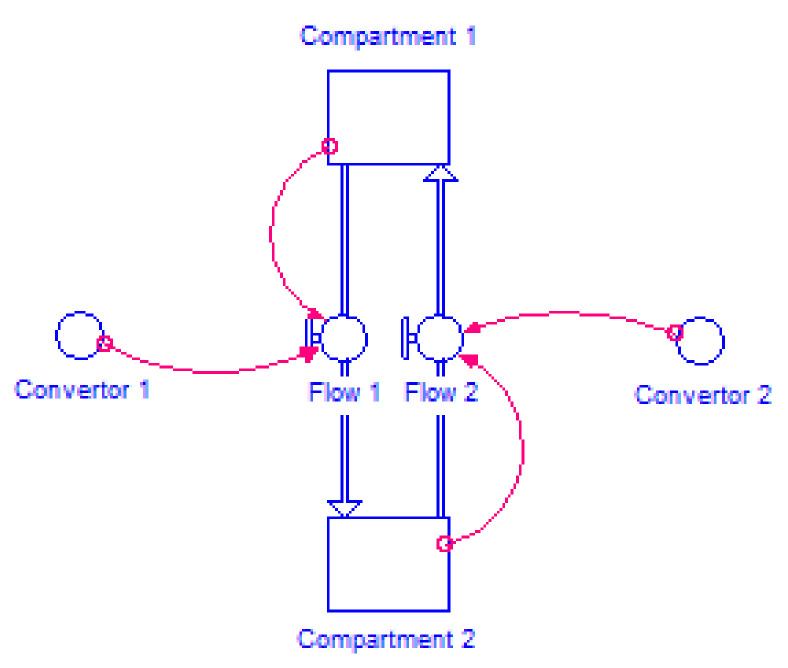
Schematic representation of a model built in STELLA^®^ modeling program. In this model, mass flows from compartment 1 to compartment 2 and from compartment 2 to compartment 1. Flow rates 1 and 2 are determined by convertor 1 and 2 respectively.

STELLA^®^ (ISEE Systems Inc., Lebanon, PA, USA) is a simulation software application that enables the study of systems through its graphical representation. The program uses Compartments, Flows, Converters, and Connectors as building blocks. “Compartments” accumulate whatever flows into them, net of whatever flows out of them, with “Flows” filling and draining accumulations. The Converter serves a utilitarian role in the software. It holds values for constants, defines external inputs to the model and calculates algebraic relationships. In general, it converts inputs into outputs. Connectors, as its name suggests, connect model elements. Moreover, the use of built-in time functions in converters, such as AND, OR, IF…THEN…ELSE or PULSE, allows a set of rules to be established, which are used by the program to control flow through the model, enabling the construction of more complex models. When the model is complete, the user has only to establish a simulation time period and a time increment (*h*). Each value calculation can be made using either Euler’s, 2nd, 3rd, or 4th order Runge-Kutta methods, being Euler’s the simplest version of the Runge-Kutta method. These are integration methods that estimate a new value (y_i+1_) through the extrapolation of an old value (y_i_) following Equation (1). In Euler’s method, φ is the slope in x_i_ (first derivate in x_i_). In the Runge-Kutta method, instead of one single derivation, one or more representative slopes (depending on the order of the method) are determined during an interval, *h*, to estimate the new value. This equation can be applied step by step and trace out the trajectory of the solution (Figure 1) [10,11,12,13].

(1) $$y_{i+1}=y_{i}+\varphi h$$

GastroPlus™ (Simulations Plus, Inc., Lancaster, CA, USA) is an advanced technology computational program used in drug R&D. Contrary to STELLA^®^, GastroPlus™ is specifically designed for PK studies, particularly physiologically based pharmacokinetics (PBPK) and physiologically based biopharmaceutics modeling (PBBM). Additionally, its incorporated absorption model, ACAT (Advanced Compartmental Absorption and Transit model), allows the simulation of intravenous, gastrointestinal, ocular, nasal, and pulmonary absorption of molecules. This enables the user to obtain a detailed absorption profile of the molecules in study, since it considers several physiological variables. GastroPlus™ simulations rely on the numerical integration of differential equations that coordinate a set of well-characterized physical events that occur and are interconnected as a result of diverse physicochemical and biologic phenomena. Furthermore, GastroPlus™ has additional modules, including ADMET Predictor™ module for the prediction of physicochemical and pharmacokinetic parameters of compounds, and other modules for deeper insight into the pharmacokinetics of a drug, such as PKPlus™ and PBPKPlus™. Despite its sophistication, GastroPlus™ is relatively easy for someone with a background in ADME to learn and use because it incorporates an intuitive and modern graphical user interface that enables a rapid and smooth transition from setting up inputs to evaluating results [14].

The lack of satisfactory results with single-agent therapy in patients led to the introduction of drug combination therapies in health care. The use of drugs with different mechanisms of action enables multiple targeting, either within the same cell or in multiple cell subpopulations, or the targeting of multiple diseases simultaneously, providing more effective treatment. This strategy’s possible favorable outcome includes the enhancement of the efficacy of the therapeutic effect, reducing the dosage, but increasing or maintaining the efficacy, minimizing or avoiding possible toxicity, and reducing or slowing down the development of drug resistance. Due to these therapeutic benefits, drug combinations have been widely used and became the leading choice for treating complicated and complex diseases, such as cancer and infectious diseases, including AIDS [15,16,17].

Several in silico tools have been developed for theoretical drug combination studies. Some research areas are more focused on predicting whether the combined effect may be synergistic, additive or antagonistic, while others are more interested in predicting drug-drug interactions. For this, several PK modeling programs, such as Simcyp^®^ (Certara UK Limited, Sheffield, UK) or GastroPlus™, now have specific modules to predict drug-drug interaction, using previous knowledge on main metabolizing enzymes of each drug in study [18,19,20]. Although a wide variety of in silico tools is already available for drug combination studies, new approaches can be proposed, and drug combination effect coupled with drug disposition simulation is an example of a gap in the existing resources.

Cancer treatment by chemotherapy is one of the most used methodologies in cancer therapy, either as primary treatment or as an adjuvant to other treatment modalities, such as surgery, radiotherapy or immunotherapy. This approach involves the use of low-molecular-weight drugs to destroy or reduce the proliferation of cancer cells [21]. This work evaluated the activity of two traditional anticancer drugs (ACDs), gemcitabine and 5-fluorouracil (5-FU), and the effect of the combination of these ACDs with repurposed drugs itraconazole, verapamil and tacrine, based on and extending previous research carried out by our group [22].

The goal of the present study was to evaluate, model and predict the performance of anticancer drugs and drug combinations in humans, through in vitro and in silico approaches. In short, the drug combinations effect was evaluated through in vitro methodologies and the results were then modeled and analyzed in silico in more detail. Inhibition of cell growth was assessed with the MTT cell viability assay in healthy and cancer human prostate cell lines (PNT2 and PC-3, respectively), and in NSCLC human cell line A549. Six different drug combinations were tested, where one of the drugs is a clinically used anticancer drug (ACD) (gemcitabine or 5-fluorouracil) and the other is a repurposed drug (RD) with promising qualities for cancer treatment (itraconazole, verapamil and tacrine). At this stage in our research, the main goal was to understand if ACDs’ activity is enhanced in the presence of one of the RD in these cell lines. Then, the aim of the in silico approach was the development of two-compartment PK models that mimic the drug combination effect previously assessed in vitro and to couple it to drug’s human PK. In sum, using multiples approaches, this work provides a better general comprehension of drug combinations’ performance in the context of cancer therapy, allowing the assessment of the PK behavior in the human body over time, and the evaluation of varying doses and its influence.

## 2. Materials and Methods

### 2.1. Chemicals

Gemcitabine, 5-FU, itraconazole, tacrine, and verapamil were obtained from Sigma-Aldrich^®^/Merck© (2021 Merck KGaA, Darmstadt, Germany) and dissolved in sterile dimethyl sulfoxide (DMSO) at 100, 50, 17, 10 and 10 µM, respectively, as stock solutions. The drugs were stored at −20 °C and diluted with culture medium prior to use.

### 2.2. Cell Culture

The normal human prostate epithelium cell line PNT-2, human prostate adenocarcinoma cell line PC-3 and human lung carcinoma cell line A549 were obtained from the American Type Culture Collection and maintained in RPMI-1640 medium with 2 mM L-glutamine (Gibco™, Thermo Fisher Scientific, Inc., Waltham, MA, USA) supplemented with 10% heat-inactivated fetal bovine serum (FBS) (Gibco™) at 37 °C in a 5% CO_2_ atmosphere.

All the cell culture procedures were carried out under sterile conditions in biological safety cabinets, using sterile reagents and materials. Cells were routinely kept exponentially growing and were sub-cultured by trypsinization twice a week. Cell viability of the cell cultures was routinely evaluated using the trypan blue exclusion assay. All experiments were carried out with exponentially growing cells having over 90% of cell viability.

### 2.3. Evaluation of Cell Growth Inhibition with the MTT Assay

#### 2.3.1. Dose-Response Curve Determination for ACDs and ACD-RD Combinations

The optimal cell concentration determined was 4 × 10^4^ cells/mL (for all the cells lines) and was then used in the MTT assays. The cells were incubated for 72 h with different concentrations of ACD or ACD-RD combination. Cells were allowed to adhere to the plate for 24 h and then 100 μL/well of drug solution were added. The multiple serial dilutions tested of each drug solution were prepared in culture medium (RPMI-1640 medium + 10% FBS). The concentrations tested ranged from 0.01 to 50 μM for gemcitabine and from 0.05 to 100 μM for 5-FU. When evaluating drug combinations, 50 μL/well of different concentrations of the ACD (gemcitabine or 5-FU) were added to the cells along with 50 μL/well of a fixed concentration of RD. The same ranges of concentrations for gemcitabine and 5-FU were tested and the chosen concentration of each RD was based on the maximum plasma concentration (C_max_) found in the literature. Since gemcitabine is a prodrug, which is phosphorylated into an active drug inside the cells, we assume this conversion is complete, and something similar for 5-FU as well. As such, verapamil, itraconazole and tacrine solutions were prepared and used in this assay with the concentrations of 1, 8.5 and 0.24 μM, respectively [23,24,25,26]. A DMSO control was also included in the experiments (maximum concentration used, 0.2%, was previously considered non-toxic to the cells). After 72-h incubation, the media was removed by aspiration, 100 μL/well of MTT solution (0.5 mg/mL in media) was added to each well and cells were incubated for another 3 h. The MTT solution was then removed by aspiration, cells were washed with 100 μL/well of PBS and 100 μL/well of DMSO were added to dissolve the formazan crystals. Absorbance was read in a spectrometer (Varioskan™ LUX multimode microplate reader) at 540 nm. Results were treated in Microsoft Excel and GraphPad Prism 6. The dose-response curves for each treatment were then plotted in appropriate graphs, differences between treatments were compared and the IC50 value, indicating the concentration resulting in inhibition of 50% of the maximal cell growth, was determined. The percentage of cell growth inhibition resulting from each drug was calculated as: [(OD 540 control cells—OD 540 treated cells)/OD 540 control cells] × 100. These assays were repeated in at least three independent experiments.

#### 2.3.2. Further Studies with Itraconazole in Combination with Gemcitabine or 5-FU

The results from the previous assay demonstrated itraconazole had the greatest ability to enhance ACDs’ activity. To study how the drug combination response is affected by itraconazole concentration, additional studies were performed. For these, only the human lung carcinoma cell line A549 was used for simplification purposes. Two studies were then performed:

1. Range of itraconazole concentrations + fixed concentration of ACD (Gem or 5-FU); itraconazole’s concentrations ranged from 0.07 to 4.25 μM, since the concentration used in the previous experiments was 8.5 μM (note dilution factor of 2 in the well). The concentration chosen for Gem and 5-FU was the one that showed the lowest effect in the previous experiments: 0.01 and 1 μM, respectively. The ACDs were also tested alone for control purposes;
2. Range of ACD (Gem or 5-FU) concentrations + fixed concentration of itraconazole (three different concentrations were tested). Different concentrations of ACD (Gem or 5-FU) were added to the wells, as well as a fixed concentration of itraconazole. The multiple serial dilutions tested for ACD ranged from 0.005 to 10 μM for Gem (since in the previous experiments the resulting dose-response curve did not have the ideal shape), and the range was maintained for 5-FU. The concentrations for itraconazole were 2, 4 and 6 μM (concentrations within the range that showed an effect when administered with a very low concentration of ACD). Itraconazole was also tested alone for control purposes.

As previously mentioned, following the 72-h cellular treatment, MTT assay was performed. These assays were repeated in at least three independent experiments each.

### 2.4. Model Development

From data available in the literature and the results of the experimental work, pharmacokinetic models were built in the simulation software STELLA^®^ 10.0.3 (ISEE Systems Inc., Lebanon, PA, USA). The structure of the model, as well as all the equations, variables and constants used for this purpose, are described in detail in the following sections.

#### 2.4.1. WinNonlin: PK Analysis

Compartmentally based PK STELLA^®^ models require the input of PK parameters of each drug, such as the volume of distribution in central and tissue compartment (V_d1_ and V_d2_, respectively), clearance (CL) and transfer rate constants from central compartment to tissue compartment and from tissue compartment to central compartment (k_12_ and k_21_, respectively). Ideally, all parameters would belong to the same source: human plasma concentration versus time data (C_p_-time data) belonging to the same ethnicity, gender, and age. Due to lack of data available in the literature concerning this issue, the only mandatory conditions were that collected data for this study was from human patients and that all the PK data for each drug belonged to the same literature source.

Phoenix WinNonlin (Certara UK Limited, Sheffield, UK) is a pharmacokinetic/pharmacodynamic (PK/PD) modeling software that, through the analysis of the C_p_-time data of a certain drug, can generate its PK parameters.

For gemcitabine, the C_p_-time data collected were from NSCLC Chinese patients [27]. Briefly, gemcitabine was intravenously infused for 120 min at a rate of 15.7 mg·min^−1^ and plasma samples were collected until 210 min after infusion start. In relation to 5-FU, C_p_-time data collected belonged to English cancer patients [28]. 5-FU was administered over 1 min, by intravenous bolus injection, at a dose of 900 mg. Plasma samples were collected for 90 min. For itraconazole, the data is relative to healthy subjects from The Netherlands, who received 100 mg administered intravenously, over 1 h, and plasma samples were collected for 96 h [29].

#### 2.4.2. Model Structure

Several models were built depending on the case study, since each drug has a specific route of administration and particular properties. The model structure differs according to the variables, constants and some equations corresponding to each case. Moreover, the drugs in each drug combination do not share metabolic pathways or transporters and only one has high protein binding. Therefore, it can be assumed that no drug interaction will occur, and each drug disposition will not be affected. Therefore, the compartmental models are developed for each drug separately, but they are connected in the same human model. The layout of each model will be detailed for each case.

#### 2.4.3. Determination of the AUC for Each Drug Combination Effect in Humans

AUC is usually calculated when bioavailability is concerned, to compare two different drug formulations, routes of administration or the effect of food on the bioavailability of a certain drug, for example [30,31]. In the models developed in this work, “AUC plasma concentration” was calculated for model validation purposes. “AUC_effect_” was determined to evaluate the overall effect of each drug combination tested in the NSCLC cell line A549. In current STELLA^®^ models, AUC is recorded using a separate compartment, but it follows a principle which is mathematically expressed in Equation (2):

(2) $$\mathrm{AUC}=\mathrm{AUC}\left(t-\mathrm{dt}\right)+“\mathrm{variable}\;\mathrm{in}\;\mathrm{study}”*\mathrm{dt}$$

The model built for gemcitabine and 5-FU is a two-compartmental model since their C_p_-time profile has a two-compartment distribution. Using gemcitabine and 5-FU C_p_-time data for intravenous infusion obtained from literature and the PK/PD modeling program Phoenix WinNonlin (64-bit, version 7.00), the curve that best fitted the experimental values obtained in the experimental in vitro approach was traced and the values for each PK parameter (V_d1_, V_d2_, CL, k_12_ and k_21_) were obtained. Therefore, with the input of the parameters obtained through WinNonlin, the models developed in STELLA^®^ describe the disposition of each drug over time, after intravenous infusion. The input dose is also the same as the one reported in the literature source used (infusion of 15.7 mg·min^−1^ for 120 min for gemcitabine, and bolus injection of 900 mg for 5-FU).

The gemcitabine and 5-FU concentration-dependent percentage of cell growth inhibition was determined by the previous in vitro studies, where a range of concentrations of either gemcitabine or 5-FU was tested. Therefore, in the models, gemcitabine/5-FU tissue concentration was linked to percentage of cell growth inhibition in cancer cells, i.e., the percentage of cell growth inhibition depends on the concentration of anticancer drug in the tissue compartment over time. Although tumor microenvironments have different characteristics from healthy tissues, including vascularization and permeability, for simplification purposes, in the developed models it was assumed that tumor tissue behaves the same way as other tissues grouped in tissue compartments.

Dose-response curves were obtained from the in vitro studies in the A549 cell line as reported above. With the constants obtained from those curves, such as the lowest and highest effect achieved (“Bottom” and “Top” respectively), the steepness of the curve (“Steepness factor”) and the drug concentration at which 50% of the maximum effect was obtained (“EC_50_”), the effect over time can be determined through a Hill Equation (3) (because tissue concentration will vary over time):

(3) $$\%\;\mathrm{of}\;\mathrm{effect}=\left(\mathrm{Bottom}\right)+\frac{\left(\mathrm{Top}\right)-\left(\mathrm{Bottom}\right)}{1+{\left(\frac{\mathrm{tissue}\;\mathrm{concentration}}{{\mathrm{EC}}_{50}}\right)}^{-\mathrm{steepness}\;\mathrm{factor}}}$$

The parameters from all the 8 dose-response curves were used in this model (one at a time) and for each, the AUC_effect_ was evaluated: Gemcitabine alone; gemcitabine + itraconazole 2 μM; gemcitabine + itraconazole 4 μM and gemcitabine + itraconazole 6 μM; 5-FU alone; 5-FU + itraconazole 2 μM; 5-FU + itraconazole 4 μM and 5-FU + itraconazole 6 μM. All the variables, such as drug plasma concentration, drug tissue concentration, drug amount eliminated, and percentage of effect can be plotted in graphs or tables and evaluated over time.

For the simulations, Runge-Kutta 4th order integration method was used since it is the most accurate integration method available in STELLA^®^. Simulation length and step size (*h*) were chosen in a way that *h* was low enough to give accurate results without compromising the speed of the simulation and simulation length was long enough to allow the lower effect value to be reached. Therefore, *h* = 0.02 and 400 min of simulation length were used for gemcitabine and *h* = 0.02 and 200 min of simulation length were used for 5-FU.

**Figure 2:**
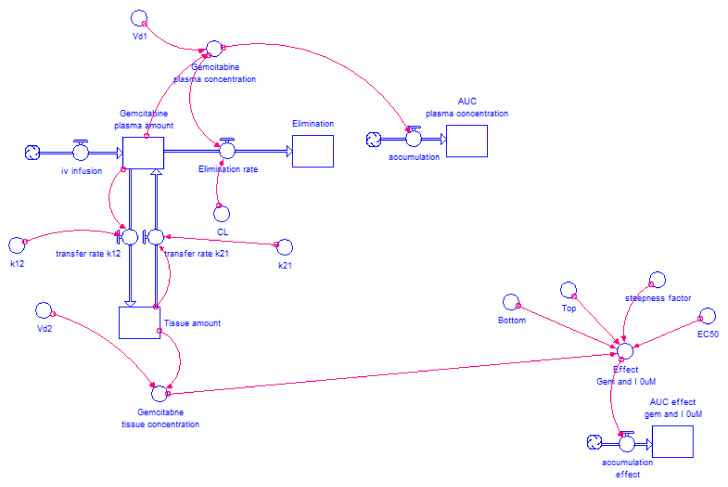
Two-compartment model of gemcitabine intravenous infusion administration. The drug is infused to plasma compartment at a rate of 15.7 mg·min^−1^ for 120 min. The drug is transferred from plasma compartment to tissue compartment and vice versa at a rate defined by “k_12_” × ”gemcitabine plasma amount” and “k_21_” × ”Tissue amount”, respectively, where “k_12_” and “k_21_” are transfer rate constants. The drug is eliminated from plasma compartment to elimination compartment at a rate defined by “CL” × ”gemcitabine plasma concentration” where “CL” is a constant and “gemcitabine plasma concentration” is a variable that changes over time. “Gemcitabine plasma concentration” is the result of “gemcitabine plasma amount” divided by “V_d1_”, while “gemcitabine tissue concentration” results from “Tissue amount” divided by “V_d2_”. “Gemcitabine plasma amount” is the net result of the amount of drug that leaves plasma compartment (to elimination and tissue compartment) and the amount that enters in this compartment (coming from the infusion and tissue compartment). “AUC plasma concentration” is generated through Equation (2), where the variable in study is “gemcitabine plasma concentration”. Considering Equation (3), “gemcitabine tissue concentration” and the four parameters obtained from gemcitabine without itraconazole dose-response curve (“Bottom”, “Top”, “Steepness factor” and “EC_50_”), the effect of gemcitabine alone is modelled over time.

**Figure 3:**
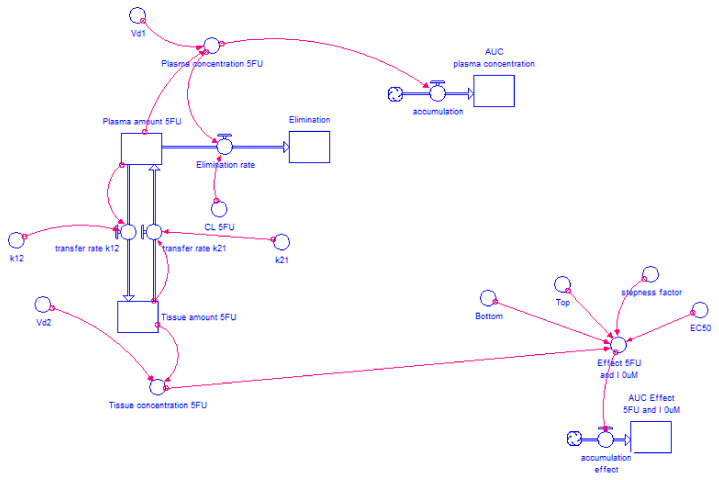
Two-compartment model of 5-FU intravenous injection administration. The drug is injected into plasma compartment at a dose of 900 mg. The drug is transferred from plasma compartment to tissue compartment and vice versa at a rate defined by “k_12_” × ”Plasma amount 5-FU” and “k_21_” × ”Tissue amount 5-FU”, respectively, where “k_12_” and “k_21_” are transfer rate constants. The drug is eliminated from plasma compartment to elimination compartment at a rate defined by “CL” × ”Plasma concentration 5-FU”, where “CL” is a constant and “Plasma concentration 5-FU” is a variable that changes over time. “Plasma concentration 5-FU” is the result of “Plasma amount 5-FU” divided by “V_d1_” while “Tissue concentration 5-FU” results from “Tissue amount 5-FU” divided by “V_d2_”. “Plasma amount 5-FU” is the net result of the amount of drug that leaves plasma compartment (to elimination” or tissue compartment) and the amount that enters in this compartment (coming from tissue compartment). “AUC plasma concentration” is generated through Equation (2) where the variable in study is “Plasma concentration 5-FU”. Considering Equation (3), “Tissue concentration 5-FU” and the four parameters obtained from 5-FU without itraconazole dose-response curve (“Bottom”, “Top”, “Steepness factor” and “EC_50_”), the effect of 5-FU alone is modeled over time.

Figure 2 and Figure 3 show the models for gemcitabine and 5-FU, respectively, without itraconazole (I = 0 μM). For the combinations of gemcitabine or 5-FU with itraconazole, the exact same layout was used and only the “Bottom”, “Top”, “Steepness factor” and “EC_50_” variables were changed, according to each situation.

#### 2.4.4. Itraconazole’s Dose-Dependent Effect, when Combined with Gemcitabine or 5-FU

After quantification of each drug combination effect (through the determination of their AUC_effect_), where the main variable was ACD tissue concentration, itraconazole’s dose-dependent effect was evaluated. At this stage, the exact same 8 dose-response curves were considered: ACD alone; ACD + itraconazole 2 μM; ACD + itraconazole 4 μM and ACD + itraconazole 6 μM. The difference from the previous study lies in the addition of a second two-compartment model (this time for itraconazole) and the itraconazole dose-dependent effect evaluation. For this purpose, three different itraconazole doses were evaluated in the simulations (100, 300 and 500 mg). ACD doses remained the same as in the previous study (1884 mg of gemcitabine and 900 mg of 5-FU).

Two-compartment PK models were built for each ACD in study, in accordance with the literature information about the model that best fits their C_p_-time data. Although itraconazole is most frequently administered orally, the intravenous infusion was selected to avoid the low oral bioavailability characteristic of this drug. Therefore, based on the literature human C_p_-time data for intravenous infusion of itraconazole and using the WinNonlin program, all PK parameters needed for two-compartment model construction were collected and the model was built.

**Figure 4:**
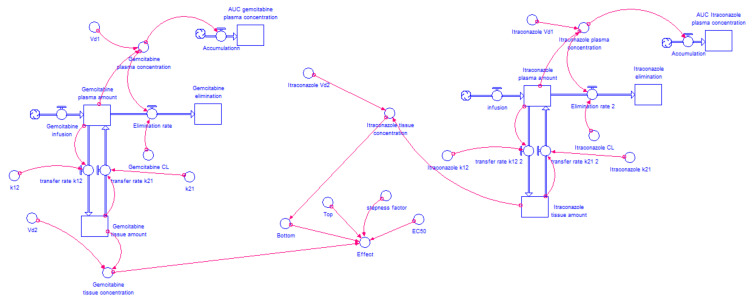
Two-compartment PK model for gemcitabine with IV infusion, two-compartment PK model for itraconazole with IV infusion and the relation of their tissue concentration with percentage of cell growth inhibition in A549 cancer cell line. Gemcitabine’s model has been described in Figure 2. For itraconazole compartmental model, drug is infused to plasma compartment at a rate of 8.3, 5 or 1.7 mg·min^−1^ during 1 h (500, 300 or 100 mg doses, respectively). It is transferred from plasma compartment to tissue compartment and vice versa at a rate defined by “k_12_” × ”Itraconazole plasma amount” and “k_21_” × ”Itraconazole tissue amount”, respectively, where “k_12_” and “k_21_” are transfer rate constants. The drug is eliminated from plasma compartment to elimination compartment at a rate defined by “CL” × ”Itraconazole plasma concentration”, where “CL” is a constant and “Itraconazole plasma concentration” is a variable that changes over time. “Itraconazole plasma concentration” is the result of “Itraconazole plasma amount” divided by “V_d1_” while “Itraconazole tissue concentration” results from “Itraconazole tissue amount” divided by “V_d2_”. “Itraconazole plasma amount” is the net result of the amount of drug that leaves plasma compartment (to elimination and tissue compartment) and the amount that enters in this compartment (coming from infusion and tissue compartment). “AUC plasma concentration” is generated through Equation (2), where variable in study is “Itraconazole plasma concentration”. Considering Equation (3), “Gemcitabine tissue concentration”, the average of gemcitabine + itraconazole dose-response curve parameters “Top”, “Steepness factor” and “EC_50_”, and Equation (4), itraconazole dose-dependent effect was modelled.

**Figure 5:**
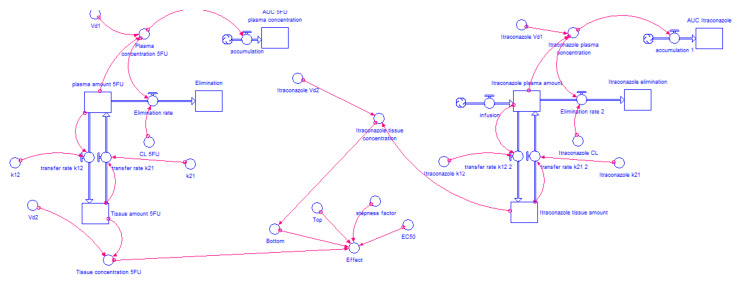
Two-compartment PK model for 5-FU with IV injection (described in Figure 3), two-compartment model for itraconazole with IV infusion and the relation of their tissue concentration with percentage of cell growth inhibition in A549 cancer cell line. For itraconazole, compartment model drug is infused to plasma compartment at a rate of 8.3, 5 or 1.7 mg·min^−1^ during 1 h (500, 300, or 100 mg doses, respectively). It is transferred from plasma compartment to tissue compartment and vice versa at a rate defined by “k_12_” × ”Itraconazole plasma amount” and “k_21_” × ”Itraconazole tissue amount”, respectively, where “k_12_” and “k_21_” are transfer rate constants. The drug is eliminated from plasma compartment to elimination compartment at a rate defined by “CL” × ”Itraconazole plasma concentration”, where “CL” is a constant and “Itraconazole plasma concentration” is a variable that changes over time. “Itraconazole plasma concentration” is the result of “Itraconazole plasma amount” divided by “V_d1_”, while “Itraconazole tissue concentration” results from “Itraconazole tissue amount” divided by “V_d2_”. “Itraconazole plasma amount” is the net result of the amount of drug that leaves plasma compartment (to elimination and tissue compartment) and the amount that enters in this compartment (coming from infusion and tissue compartment). “AUC plasma concentration” is generated through Equation (2) where variable in study is “Itraconazole plasma concentration”. Considering Equation (3), “Tissue concentration 5-FU”, the average of 5-FU + itraconazole dose-response curve parameters “Top”, “Steepness factor” and “EC_50_”, and Equation (5), itraconazole dose-dependent effect was modelled.

Only the “Bottom” value of the ACD dose-response curve was found to be significantly affected by itraconazole concentration. Therefore, equations that relate the change of “Bottom” value with the concentration of itraconazole were included in “Bottom” Converter, i.e., the percentage of cell growth inhibition is given taking into account not only ACD concentration (Equation (3), but also the influence of itraconazole concentration on “Bottom” value. Equation (4) describes the change of “Bottom” value in gemcitabine + itraconazole dose-response curves and Equation (5) describes it for the 5-FU + itraconazole combination. *x* is itraconazole tissue concentration, in μg·mL^−1^. In relation to the other variables that describe dose-response curve (“Top”, “Steepness factor” and “EC_50_”), the average of the three values (relative to ACD + itraconazole 2, 4 and 6 μM dose-response curves) were used. The model for gemcitabine + itraconazole combination is shown in Figure 4. 5-FU + itraconazole combination model is shown in Figure 5. Again, the exact same layout was used to test three different doses of itraconazole, and 5-FU dose remained the same. All the variables, such as drug plasma concentration, drug tissue concentration, drug amount eliminated, and percentage of effect can be plotted in graphs or tables and evaluated over time. Similar Runge-Kutta 4th order integration methods were used.

(4) $$\mathrm{Bottom}=2.44x^{2}-1.95x-1.06$$

(5) $$\mathrm{Bottom}=2.15x^{2}-1.15x-0.47$$

### 2.5. Model Validation

Two assessments were carried out to establish whether two-compartment models were well-constructed and that there were no errors in parameter inputs. First, STELLA^®^ generated C_p_-time curves were plotted against literature C_p_-time data and general shape of the curve and fitting was evaluated. AUC plasma concentration was another parameter used to evaluate the accuracy of the models. Therefore, using the exact same dosages and routes of administration as the ones used in the literature experiments, C_p_-time curve and AUC plasma concentration were determined and compared with literature data.

The results of the present study provide new insight in ACD and RD combinations evaluated for lung and prostate cancer treatment and a new tool to quantify drug combinations effect, as the area under the dose-response-time curve, or AUC_effect_. Furthermore, the innovative idea developed in this work resides in an in silico study that enables the coupling of cell viability assay data with human drug disposition.

## 3. Results and Discussion

### 3.1. In Vitro Experiments—Evaluation of Inhibition of Cell Growth

To evaluate the effect of six drug combinations (ACD gemcitabine or 5-FU with RD itraconazole, verapamil or tacrine) in cell proliferation, MTT assay was performed following 72-h treatment in human lung carcinoma A549 cell line, human prostate adenocarcinoma PC-3 cell line and normal human prostate epithelium PNT2 cell lines.

#### 3.1.1. Range of ACD Concentrations + Fixed Concentration of RD

**Figure 6:**
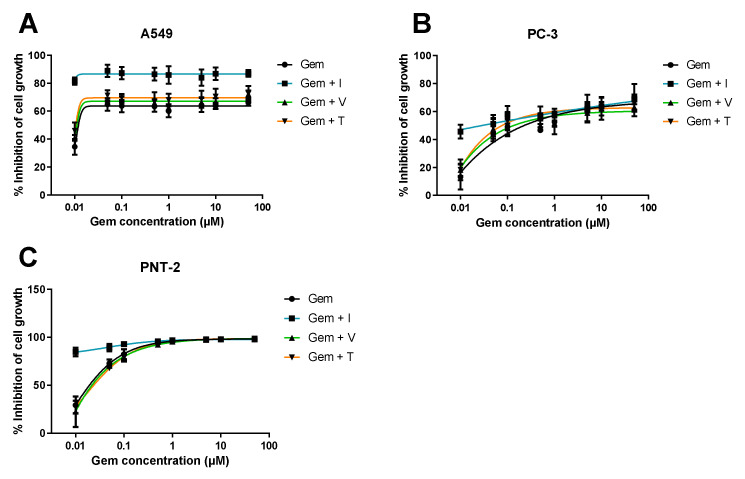
Dose-response curve of gemcitabine in combination with RD. Percentage of cell growth inhibition of human lung carcinoma A549 cell line ((**A**)—top left), human prostate adenocarcinoma PC-3 cell line ((**B**)—right top) and normal human prostate epithelium PNT2 cell line ((**C**)— bottom left), treated with a wide range of concentrations of gemcitabine (Gem) alone (black line) or Gem in combination with a fixed concentration of RD (verapamil, V, green line; itraconazole, I, blue line; or tacrine, T, orange line), during 72 h, determined with MTT assay. The results are the mean of at least three independent experiments. The DMSO control did not present toxicity to the cells (data not shown).

**Figure 7:**
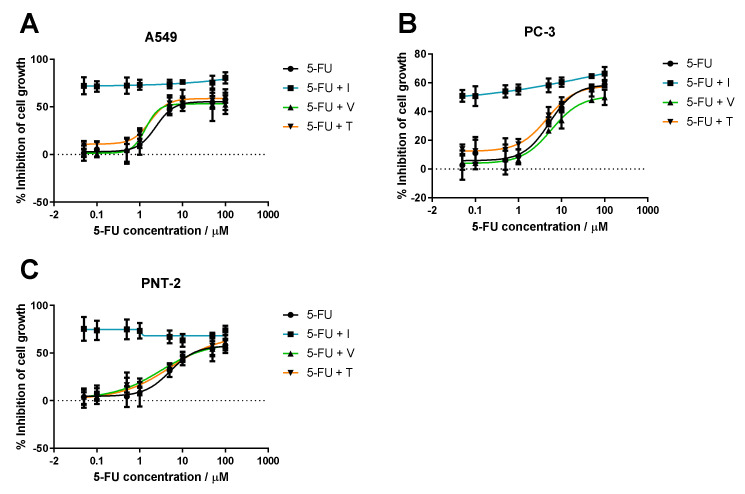
Dose-response curve of 5-FU in combination with RD. Percentage of cell growth inhibition of human lung carcinoma A549 cell line ((**A**)—top left), human prostate adenocarcinoma PC-3 cell line ((**B)**—top right) and normal human prostate epithelium PNT2 cell line ((**C**)—bottom left), treated with wide range of concentrations of 5-fluorouracil (5-FU) alone (black line) or 5-FU in combination with a fixed concentration of RD (verapamil, V, green line; itraconazole, I, blue line; or tacrine, T, orange line), during 72 h, determined with MTT assay. Results are the mean of at least three independent experiments. The DMSO control did not present toxicity to the cells (data not shown).

Firstly, several ACD concentrations (gemcitabine or 5-FU) were tested with or without RD (itraconazole, verapamil or tacrine) at a fixed concentration. The dose-response curves are represented in Figure 6 and Figure 7.

These results show all tested cell lines are sensitive to gemcitabine and 5-FU but to different degrees. The dose-response curve for gemcitabine and 5-FU alone is approximate to gemcitabine and 5-FU in combination with verapamil or tacrine, in all cell lines. On the other hand, itraconazole combination substantially improved the overall effect, comparing to ACD alone. In healthy and cancer prostate cell lines (PNT2 and PC-3, respectively), itraconazole effect is more noticeable when gemcitabine concentration is low (<0.5 μM in PNT2 and <0.1 μM in PC-3 cell lines). At higher concentrations, Gem + I curve matches the control line, suggesting that the overall effect is due to gemcitabine. In the A549 cell line, itraconazole considerably enhanced cell growth inhibition in all gemcitabine concentrations tested. However, the overall effect of 5-FU + I combination is almost independent on 5-FU concentration, suggesting that the observed response is mostly due to itraconazole. Yet, this conclusion cannot be validated due to the lack of itraconazole alone control.

The three selected repurposed drugs were expected to show noticeable improvement in cell growth inhibition when combined with gemcitabine or 5-FU. However, tacrine and verapamil did not reveal promising activity. Tacrine has been reported to enhance tumor suppressor’s activity, such as caspase, Bax, and p53 expression in mouse hepatocytes [32]; though, when used in combination with gemcitabine or 5-FU at a concentration of 1 μM in the cell lines tested here, it did not reveal significant improvement in cell growth inhibition comparing to the control (ACD alone). Verapamil is known to promote intracellular accumulation of chemotherapeutic drugs. It has been studied in multiple types of cancer cell lines and in combination with multiple ACDs and has proven efficacy in reversing multidrug resistance through inhibition of P-gp, one of the main proteins responsible for drug extrusion in cancer [31,33,34,35,36,37]. A recent study has also evaluated the combination of verapamil with gemcitabine in chemotherapy-resistant pancreatic cancer side population (SP) cells, showing enhancement in cytotoxicity when used in combination with this chemotherapeutic agent, as well as apoptosis induction of stem-like SP cells in L3.6pl and AsPC-1 pancreatic carcinoma models and significant inhibition of pancreatic cancer tumor growth in vivo, potentially by targeting stem-like side population cells [38]. However, in this work, verapamil did not show significant improvement of cell growth inhibition when in combination with gemcitabine or 5-FU.

As shown in the results above, itraconazole was the only RD that significantly increased the cell growth inhibition when combined with an ACD (gemcitabine or 5-FU). Itraconazole has been extensively studied in cancer research and several anticancer mechanisms of action have been identified, which include the ability to inhibit Hedgehog pathway and angiogenesis, both mechanisms related to cancer development, autophagy induction and reversal of multidrug resistance [39,40,41,42,43,44]. Therefore, the enhancement of cell growth inhibition may be explained by the targeting of an additional pathway in cell division. To better understand how the itraconazole concentration affected cell growth inhibition, lower itraconazole concentrations were tested, since the concentration tested in the previous experiments was the maximum plasma concentration recommended.

#### 3.1.2. Range of Itraconazole Concentrations + Fixed Concentration of ACD

**Figure 8:**
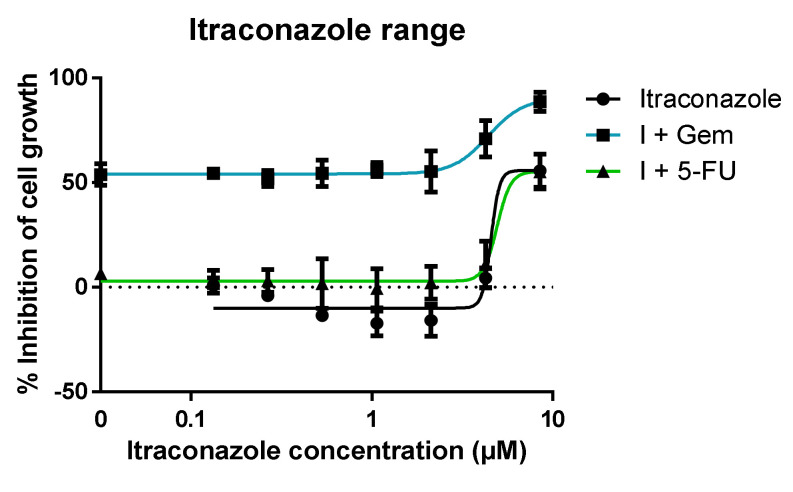
Dose-response curve of itraconazole in combination with ACD. Percentage of cell growth inhibition of human lung carcinoma A549 cell line treated with a range of concentrations of itraconazole alone (black line) or itraconazole in combination with a fixed concentration of ACD (Gem (blue line) or 5-FU (green line)), during 72 h, determined with MTT assay. The results are mean of at least three independent experiments. The DMSO control did not present toxicity to the cells (data not shown).

A range of concentrations of itraconazole was either tested alone (control) or in combination with a fixed concentration of ACD. ACDs were also tested alone as a control. Itraconazole dose-response curve is represented in Figure 8, demonstrating no cell growth inhibition is achieved at concentrations lower than 2 μM. Concentrations higher than this value drove cell growth inhibition in a dose-dependent manner. However, the dose-response curve of itraconazole + 5-FU overlaps itraconazole alone dose-response curve. Hence, this data is not enough to understand if itraconazole is improving 5-FU effect or if the result is only due to itraconazole itself. For the itraconazole + gemcitabine combination, at higher concentrations of itraconazole the effect is higher than itraconazole or gemcitabine effect alone. As stated, only itraconazole concentrations above 2 μM enhanced the cell growth inhibition when in combination with an ACD. Thus, to evaluate the itraconazole concentration effect in ACD dose-response curve, further experiments were done.

#### 3.1.3. Range of ACD Concentrations + Fixed Concentration of Itraconazole

**Figure 9:**
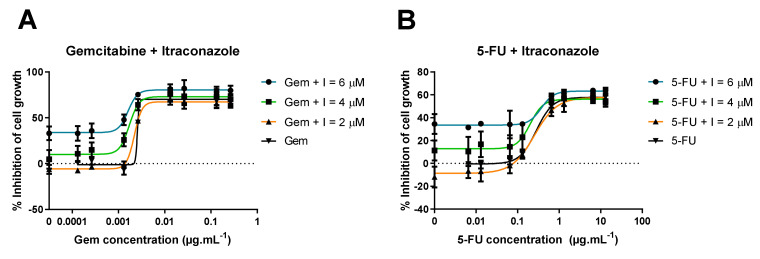
Dose-response curves of ACD in combination with itraconazole. (**A**): Percentage of cell growth inhibition of human lung carcinoma A549 cell line treated with wide range of concentrations of gemcitabine (Gem) alone (control) or Gem in combination with a fixed concentration of itraconazole (I), during 72 h, determined with MTT assay; (**B**): Percentage of cell growth inhibition of human lung carcinoma A549 cell line, treated with wide range of concentrations of 5-FU alone (control) or 5-FU in combination with a fixed concentration of I, during 72 h, determined with MTT assay. The results are the mean of at least three independent experiments. The DMSO control did not present toxicity to the cells (data not shown).

ACD range of concentrations was tested with or without a fixed concentration of itraconazole. Three different concentrations of itraconazole were tested: 2, 4 and 6 μM. Itraconazole was also tested alone as a control. Figure 9 shows gemcitabine + itraconazole (A) and 5-FU + itraconazole (B) dose-response curves. According to the results, itraconazole concentration does not significantly affect the highest percentage of cell growth inhibition (“Top” value), but it strongly affects the lowest value of the curve (“Bottom” value), meaning the highest effect (“Top” value) achieved with gemcitabine or 5-FU alone is approximately the same as drug combinations, values rounding 73% and 59%, respectively (Table 1 and Table 2). On the other hand, the lowest percentage of cell growth inhibition (“Bottom” value) strongly depends on itraconazole concentration, varying in a dose-dependent manner. Increasing itraconazole concentration to 4 and 6 μM increases “Bottom” value from 0 (control) to about 13%, and 33%, respectively, in both combination groups (Figure 9A,B).

### 3.2. WinNonlin: PK Analysis

**Figure 10:**
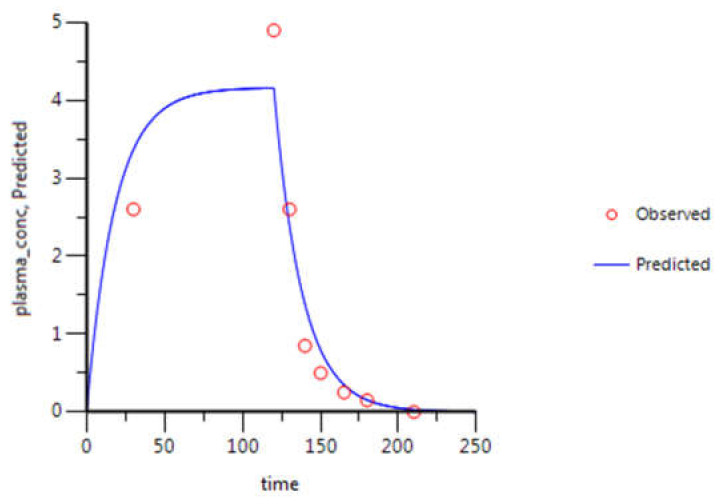
Gemcitabine C_p_-time curve prediction through two-compartment model fitting of its observed C_p_-time data. Red circles correspond to the experimental data, obtained from the literature, and the continuous blue line corresponds to the in silico C_p_-time curve prediction. Plasma concentration is given in μg·mL^−1^ and time in minutes.

The concentration-time curves of gemcitabine in plasma from the literature were evaluated by compartmental analysis (Phoenix WinNonlin (64-bit, version 7.00)). The best fitting was achieved with a two-compartmental model (Figure 10) and PK parameters were obtained through PK analysis (Table 3). Although WinNonlin prediction (blue line) did not fit all experimental values (red circles), k_10_, AUC, C_max_ and CL values obtained are in accordance with the literature. However, transfer rate constants k_12_ and k_21_ and tissue compartment volume of distribution (V_d2_) measurements are not very precise, but since no more accurate data was available, the values were included in the STELLA^®^ model.

**Figure 11:**
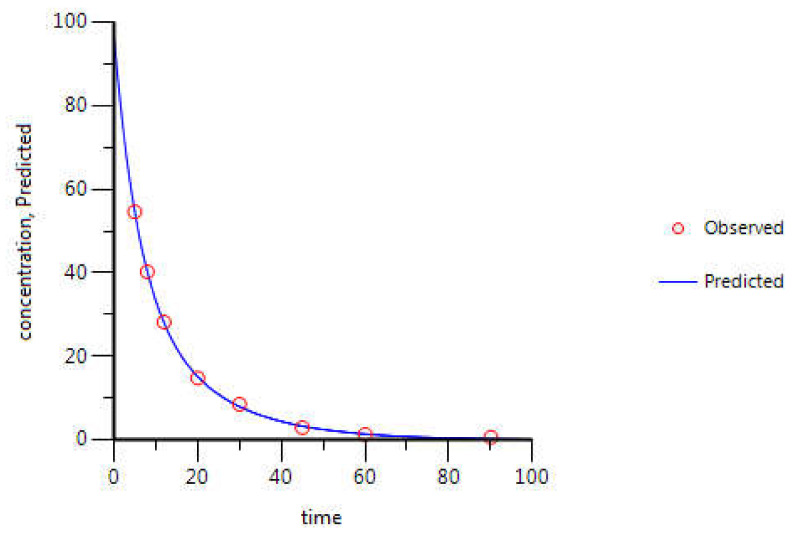
5-FU C_p_-time curve prediction through two-compartment model fitting of its observed C_p_-time data. Red circles correspond to the experimental data, obtained from the literature, and the continuous blue line corresponds to the in silico C_p_-time curve prediction. Plasma concentration is given in μg·mL^−1^ and time in minutes.

The concentration-time curves of 5-FU in plasma from the literature were also evaluated by compartmental analysis and the best fitting was achieved with a two-compartment model (Figure 11). PK parameters were obtained through PK analysis (Table 4). As shown in Figure 11, WinNonlin prediction (blue line) fits all experimental values (red circles) almost perfectly. All PK parameters obtained are also in accordance with literature values.

**Figure 12:**
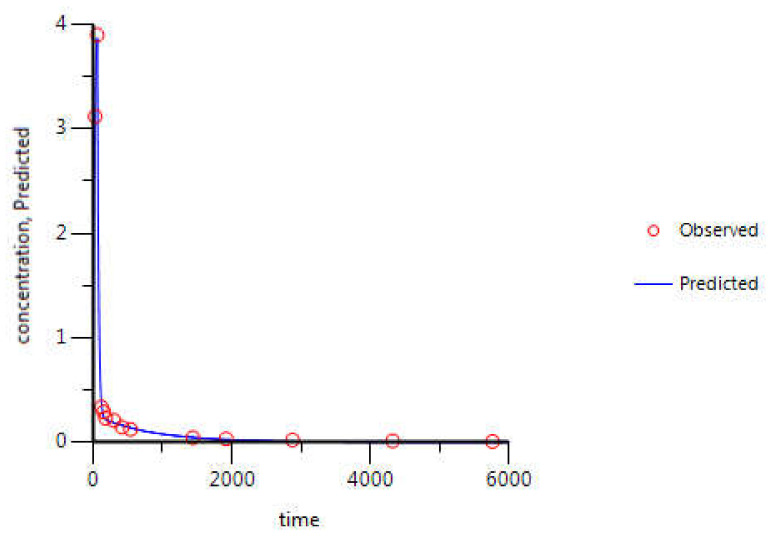
Itraconazole C_p_-time curve prediction through two-compartmental model fitting of its observed C_p_-time data. Red circles correspond to the experimental data, obtained from the literature, and the continuous blue line corresponds to the in silico Cp-time curve prediction. Plasma concentration is given in μg·mL^−1^ and time in minutes.

Finally, concentration-time curves of itraconazole in plasma were also evaluated and best-fitted to a two-compartmental model (Figure 12 and Table 5). Although the predicted steady-state volume of distribution (V_ss_) is quite lower than the volume of distribution reported in literature source, WinNonlin prediction (blue line) fits all experimental values (red circles) almost perfectly and % CV is fairly small in all parameters determined. Therefore, WinNonlin prediction was assumed to be reliable. In fact, the volume of distribution parameter determined in the literature is V_darea_ [28], which means that it was determined during the elimination phase and not at steady-state, as in WinNonlin prediction [45]. Therefore, the parameters cannot be compared.

### 3.3. STELLA^®^ Models

#### 3.3.1. Input Data for the Model

For the models described in the experimental section, two types of parameters were used: (1) the parameters related with the drug in focus obtained through WinNonlin (Table 3, Table 4 and Table 5), which were used as constants without further modification, and (2) the parameters obtained from the in vitro studies.

#### 3.3.2. Model Validation

**Figure 13:**
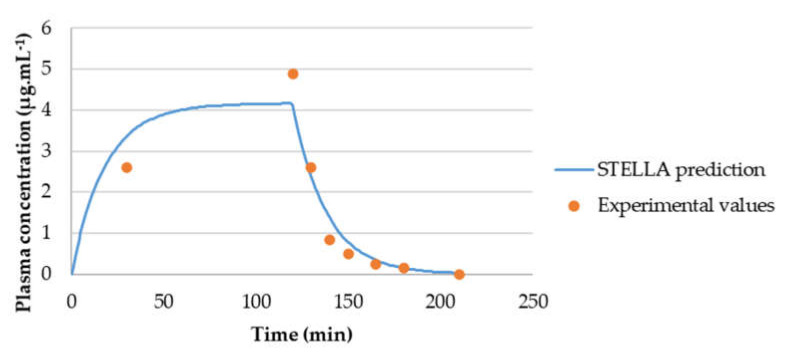
Graphical representation of experimental C_p_-time data of gemcitabine and C_p_-time curve generated in silico for this drug over 210 min.

**Figure 14:**
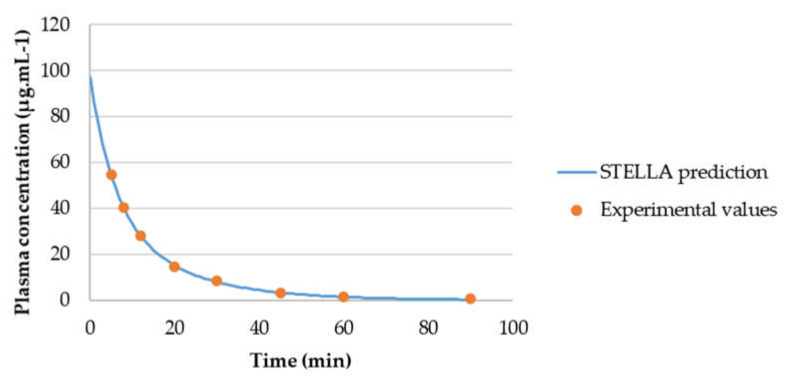
Graphical comparison between experimental C_p_-time data of 5-FU and C_p_-time curve generated in silico for this drug over 90 min.

**Figure 15:**
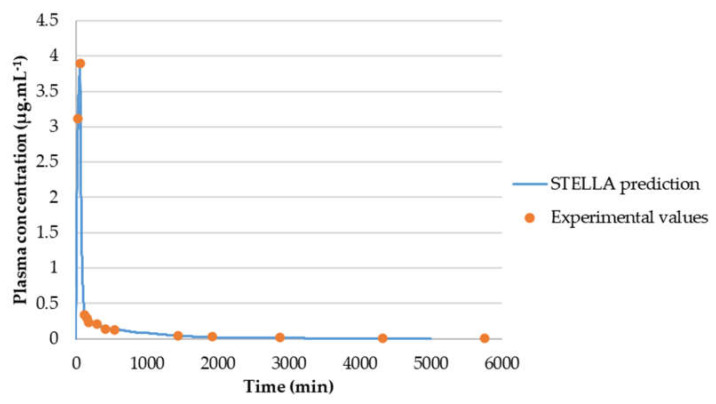
Graphical comparison between experimental C_p_-time data of itraconazole and C_p_-time curve generated in silico for this drug over 5000 min.

**Figure 16:**
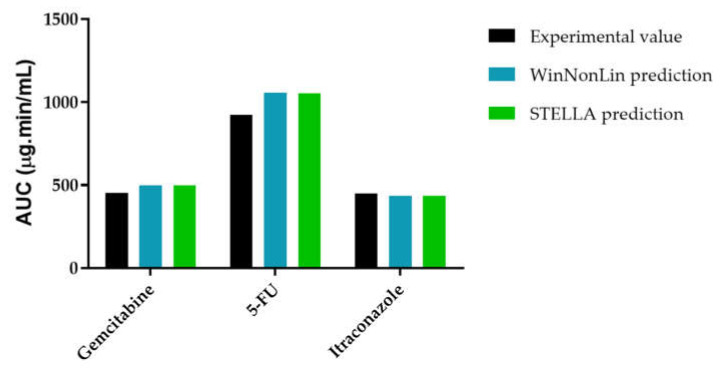
Graphical representation of AUC plasma concentration of gemcitabine, 5-FU, and itraconazole when determined experimentally, through WinNonlin or STELLA^®^ models.

To evaluate the model accuracy, C_p_-time curve was determined through STELLA^®^ models and compared with the experimental values. Figure 13 shows that gemcitabine STELLA^®^ model (Figure 2) is quite accurate in predicting gemcitabine’s plasma concentration over time. Since the input values came from WinNonlin, and WinNonlin C_p_-time curve prediction did not fit all experimental values, then C_p_-time curve predicted through STELLA^®^ will not fit them all either. For the 5-FU STELLA^®^ model, Figure 14 demonstrates C_p_-time curve predicted with STELLA^®^ (blue line) fitting all the experimental values (orange circles). Thus, model accuracy predicting 5-FU’s plasma concentration over time can be assumed. Similarly, itraconazole C_p_-time curve predicted with STELLA^®^ (blue line) is also fitting all the experimental values (orange circles) (Figure 15). Once more, model accuracy predicting itraconazole plasma concentration over time can be assumed. Besides C_p_-time curve graphical analysis, to validate STELLA^®^ models, AUC values were determined and compared with literature sources. In Figure 16, three AUC values are depicted for each drug: AUC calculated from experimental data (literature value), WinNonlin PK analysis, and STELLA^®^ model simulation prediction. As expected, for each drug, STELLA^®^ prediction is in perfect accordance with WinNonlin PK analysis, showing the exact same AUC value. Literature values are slightly different from STELLA^®^ and WinNonlin predictions, probably due to differences in the integration method used for AUC calculation (all literature sources used trapezoidal rule, while STELLA^®^ and WinNonlin predictions resorted to 4th order Runge-Kutta method).

#### 3.3.3. AUC_effect_: Drug Combination Effect Comparison

**Figure 17:**
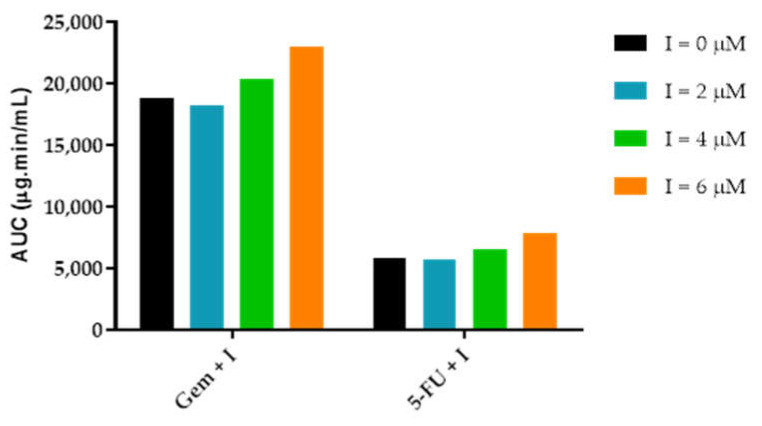
Graphical representation of AUC_effect_ for gemcitabine + itraconazole and 5-FU + itraconazole combinations.

To compare drug combination effect in A549 cancer cell line, AUC_effect_ was determined in STELLA^®^. The effect is calculated through Equation (3), where the only variable is ACD tissue concentration. All the other parameters are constants and characterize the dose-response curve obtained from the in vitro studies, i.e., depending on ACD tissue concentration and the parameters introduced in model converters, “Effect” gets a certain value over time. AUC_effect_ quantifies the overall effect during simulation. According to the simulations (Figure 17), and in accordance with the in vitro experimental results (Figure 9), the higher the itraconazole concentration, the higher is the AUC_effect_ value. In gemcitabine combinations, when itraconazole tissue concentration is 4 μM and 6 μM, AUC_effect_ is about 9% and 22% higher than control (gemcitabine without itraconazole), respectively. In 5-FU combinations, these values reach 12% and 34% improvement relative to control (5-FU without itraconazole), respectively. However, for unknown reasons, when itraconazole concentration is 2 μM, AUC_effect_ is lower than control, in both combination groups.

Although gemcitabine and 5-FU elimination half-life (t_1/2_) is identical (10 and 12 min, respectively) [27,29], the former is infused at a rate of 15.7 mg per minute, over 2 h, which represents a total dose of 1884 mg, while the latter is administered through a bolus IV injection at a dose of 900 mg. Therefore, the AUC_effect_ of 5-FU is expected to be much smaller than gemcitabine’s, due to the lower dose and overall reduced exposure time of 5-FU in tissue. Besides the AUC_effect,_ further analysis was done regarding itraconazole dose-dependent effect. This time, instead of only one variable (ACD concentration), as in the previous study, the percentage of effect will also depend on itraconazole tissue concentration over time. “% Effect” is still calculated through Equation (3), where ACD tissue concentration is the main variable, but “Bottom” parameter is now an equation dependent on itraconazole tissue concentration, instead of being a constant (Equations (4) and (5)).

**Figure 18:**
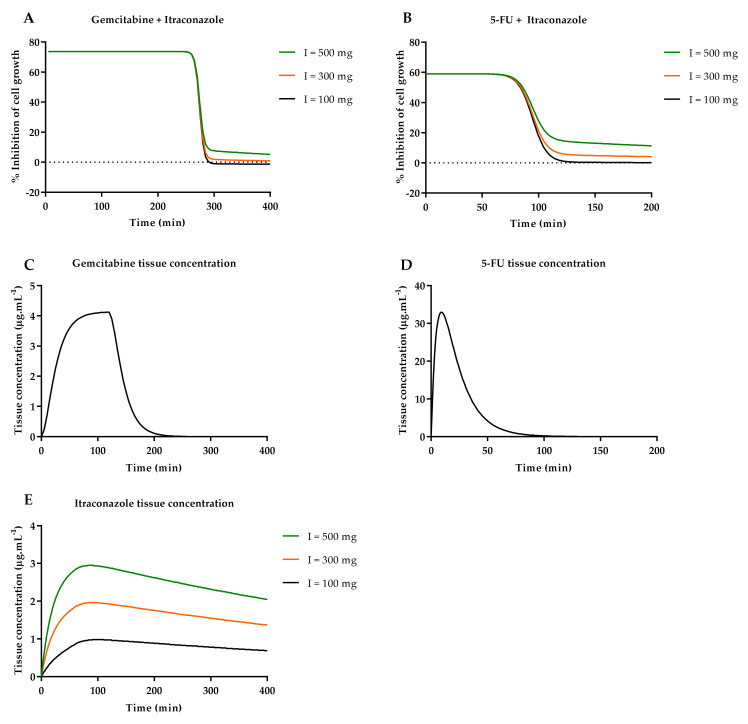
ACD + itraconazole (I) combination STELLA^®^ simulation. (**A**,**B**): Effect curves for gemcitabine + itraconazole and 5-FU + itraconazole combinations, respectively. Three itraconazole doses were tested, (**C**,**D**): Tissue concentration-time curves of gemcitabine and 5-FU, when intravenously administered at a dose of 1884 mg (infusion), and 900 mg (injection), respectively; (**E**): Tissue concentration-time curve of itraconazole for three different doses of intravenous infusion.

Figure 18 presents five different graphs that enable the evaluation of drug concentration in the tissue compartment and its relationship with % effect over time. Graphs A and B show effect-time curve of ACD and itraconazole drug combination. Using constant ACD dose administration and three different doses of itraconazole, differences between effect-time curves can be observed. According to Figure 18A,B, depending on itraconazole dose administration, the final part of the curve is different.

For gemcitabine + itraconazole drug combination (Figure 18A), first the “% Effect” remains constant, at a level of 73% of cell growth inhibition. Then, at minute 260, the effect starts dropping abruptly. This drop can be explained with a deeper analysis of Equation (3). Gemcitabine concentration affects “% Effect” through exponential function described by Equation (6), where *x* is gemcitabine tissue concentration. According to this equation, at very high concentrations, gemcitabine tissue concentration influence on “% Effect” can be despised because f(*x*) will result in a very low value (Equation (6). Then, this value will be summed to 1 and divided to (“Top”-“Bottom”) values. When this concentration is reduced to a value lower than 0.008 μg·mL^−1^, f(*x*) increases exponentially, reducing “% Effect” abruptly. Figure 18C shows gemcitabine tissue concentration-time curve:

(6) $$f\left(x\right)={\left(\frac{x}{0.0019}\right)}^{-4.67}.$$

After the drop in “% Effect” value, slight differences between effect-time curves start to be noticed. At this point, itraconazole tissue concentration plays the main role in the overall effect, since “% Effect” equals “Bottom” value (Equation (3), which is directly dependent on itraconazole tissue concentration. In Figure 18E, itraconazole tissue concentration is shown for the three studied doses. According to the results, itraconazole is slowly eliminated from the tissue compartment and will maintain % of cell growth inhibition relatively constant while it is being eliminated from the tissue compartment. In fact, if higher values of itraconazole tissue concentration were considered, “% Effect” would be equally higher. This can be mathematically explained through Equation (4) analysis. Itraconazole multiple dosing simulation was attempted in the STELLA^®^ simulation program, with the objective of increasing itraconazole tissue concentration, but limitations in the software’s built-in functions did not allow the study.

For 5-FU + itraconazole drug combination (Figure 18B), first “% Effect” remains constant, at a level of 59% of cell growth inhibition. Then, at minute 70, effect starts dropping abruptly. This drop can be explained with the same reasoning as presented above for gemcitabine (Equation (7). When 5-FU concentration is reduced to a value lower than 0.5 μg·mL^−1^, f(*x*) increases exponentially reducing “% Effect” abruptly. Figure 18D shows 5-FU tissue concentration-time curve. When the drop in “% Effect” value starts, slight differences between effect-time curves start to be noticed. At this point, itraconazole tissue concentration plays the main role in the overall effect, since “% Effect” equals “Bottom” value (Equation (3), which is directly dependent on itraconazole tissue concentration. As stated above, if higher values of itraconazole tissue concentration were considered, “% Effect” would be equally higher. This can be mathematically explained through Equation (5) analysis:

(7) $$f\left(x\right)={\left(\frac{x}{0.28}\right)}^{-2.62}$$

While drug dose-response curves enable the establishment of a relationship between drug concentration and % of cell growth inhibition, this kind of approach facilitates the study of drug concentration-% of cell growth inhibition relationship over time, providing a better understanding about for how long a drug will exert its effect when administered at a certain dose until metabolization reduces drug concentration to a non-therapeutic level.

## 4. Discussing the Limitations in Pharmacokinetics Modeling

Despite the overall success of this project, some difficulties were detected during its development, particularly regarding STELLA^®^ simulation program, namely the impossibility to make multiple dosing regimens (for IV infusion). As mentioned in Section 3.3.3., itraconazole concentration in the tissue compartment was not high enough to significantly influence the overall “% Effect”. The idea of using multiple dosing regimen was to reach steady-state plasma concentration (C_ss_), increasing itraconazole accumulation in tissue compartment, and thus, to predict the influence of itraconazole in cell growth inhibition. Thus, alternatives to the STELLA^®^ simulation program were explored to overcome this problem, which included the use of GastroPlus™ PBPK simulation software and Microsoft Excel.

**Figure 19:**
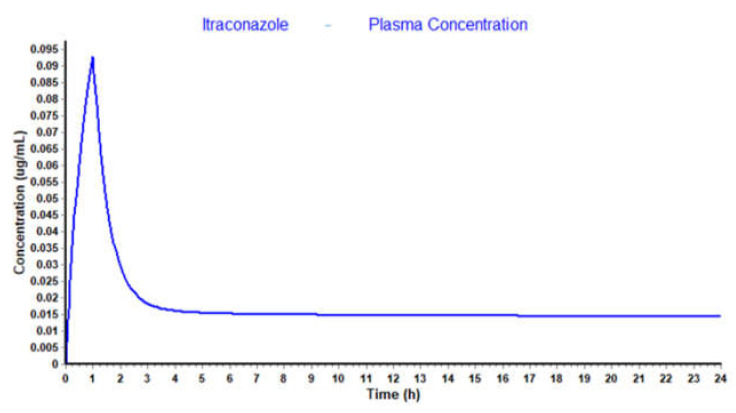
Itraconazole C_p_-time profile predicted through GastroPlus™ simulation software.

In this project, an attempt to replicate experimental C_p_-time data of itraconazole in GastroPlus™ was made, but neither uploading itraconazole molecular structure nor inputting experimental parameters could replicate the concentration plasma profile reported in the literature. As shown in Figure 19, itraconazole C_max_ predicted through this program, for 100 mg, 1 h IV infusion, is about 0.095 μg·mL^−1^, while the equivalent value reported in the literature, for the same dosing regimen, is 3.9 μg·mL^−1^. GastroPlus™ is a complex software and it is not solely ruled by simple pharmacokinetics equations. To run a simulation in this program, the input of a few parameters is needed. Apart from common parameters input as dose, dosage form, solubility and the pH at which it was measured, logP and pKa’s (if any), it also requires some knowledge about particle radius, particle density and diffusion coefficient. In the simulation presented in Figure 19, most of the parameters used were predicted through itraconazole structure upload. Even with the input of some experimental values, itraconazole C_p_-time is quite different from the reported one. Thus, it was impossible to validate the model and multiple dosing regimen could not be evaluated.

Given the circumstances, the study of itraconazole’s multiple dosing regimen was done in Microsoft Excel. The literature C_ss_ value was used as the itraconazole plasma concentration. The transfer rate constants k_12_ and k_21_ previously obtained through WinNonlin were used to simulate itraconazole flow between plasma and tissue compartment. Then, the “Bottom” value was calculated at every time point, which is dependent on itraconazole tissue concentration on that specific time point (Equation (4) or Equation (5)). Finally, “% Effect” was calculated through Equation (3).

There are several potential benefits in employing in silico models in the process of drug R&D. However, reliable results require complex and data-intensive models. Furthermore, the use of complex models in drug development requires adequate resources and well-qualified researchers with a good understanding of the ADME data required to drive the models.

STELLA^®^ models developed in this work are simple but innovative, and provided insight on the PK and effect of combinations of anticancer drugs with repurposed drugs. Ideally, parameters used in the model structure should be more consistent, but for a first idea of the general behavior of the drug combinations in human body, the data used is fairly appropriate. There is variability in the populations originating the data, and moreover, the tumor is assumed to behave like the tissues grouped into a tissue compartment, but no such assumption was confirmed or validated. Although in vitro results do not correlate directly with in vivo effect, these preliminary studies might be useful for comparative effect purposes and to provide mechanistic predictions of dosing regimens.

## 5. Conclusions

We know that the interaction of two drugs in a combination can generate distinct but complementary cellular responses, but the outcome of this study suggests an interesting hypothesis to be tested in the clinic, and the preclinical model developed in our study might provide a reference for the dose selection in the combination therapy of cancer treatment. Therefore, it is now possible to study tissue drug concentration—% of cell growth inhibition relationships over time. This provides a better understanding regarding how long a drug will exert its effect when administered at a certain dose until metabolization reduces drug concentration to a non-therapeutic level.

In the future, other drug combinations can be tested in cell models, and % effect can be evaluated over time through similar models. However, due to some limitations and inconsistencies found in the models developed here, some upgrading/alterations may be required. Furthermore, future experiments may need different model construction, depending on the context in which they are inserted, but understanding how STELLA^®^ modeling program works, one can collect and analyze the necessary data and build the most convenient model. Additionally, the effect of these combinations must be studied using appropriate in vivo models.

## Figures and Tables

**Table 1:** Dose-response curve parameters for gemcitabine and itraconazole combinations, obtained from GraphPad. (N.A.: non-applicable.).

Gem + I ^a^	Bottom (%)	Top (%)	Steepness Factor	EC_50_ (μg·mL^−1^)
I = 6 μM	34.33 ± 2.43	80.39 ± 1.71	4.30 ± 1.30	0.0016 ± 3.2 × 10^−7^
I = 4 μM	12.83 ± 2.79	73.07 ± 1.97	4.37 ± 0.99	0.0018 ± 3.1 × 10^−7^
I = 2 μM	−5.51 ± 1.83	67.47 ± 1.29	5.35 ± 1.12	0.0022 ± 1.6 × 10^−7^
I = 0 μM	−1.06 ± 2.35	70.12 ± 1.67	23.14 ± 4.20 × 10^5^	0.0026 ± 2.2 × 10^5^
Average	N.A.	72.76	4.67	0.0021

^a^ I is the drug itraconazole. Gem + I = 2 μM for “Bottom (%)” and Gem + I = 0 μM for “Steepness factor” were not included in the calculation of the average.

**Table 2:** Dose-response curve parameters for 5-FU and itraconazole combinations, obtained from GraphPad. (N.A.: non applicable.).

5-FU + I ^a^	Bottom (%)	Top (%)	Steepness Factor	EC_50_ (μg·mL^−1^)
I = 6 μM	33.22 ± 1.58	63.24 ± 1.62	2.77 ± 1.23	0.36 ± 0.01
I = 4 μM	13.42 ± 3.09	56.05 ± 2.87	2.90 ± 2.40	0.20 ± 0.0071
I = 2 μM	−6.99 ± 2.14	57.52 ± 2.11	1.70 ± 0.26	0.27 ± 0.002
I = 0 μM	−0.47 ± 1.32	57.80 ± 1.32	2.19 ± 0.27	0.28 ± 0.001
Average	N.A.	58.65	2.39	0.28

^a^ I is the drug itraconazole. 5-FU + I = 2 μM for “Bottom (%)” was not included in the calculation of the average.

**Table 3:** Gemcitabine PK parameters obtained from WinNonlin.

Gemcitabine Parameters	Estimate	CV (%)	Literature Values [27]
k_10_ (min^−1^)	5.54 × 10^−2^	144.9	7.00 × 10^−2^
k_12_ (min^−1^)	6.64 × 10^−4^	45,998.6	-
k_21_ (min^−1^)	1.02 × 10^−1^	29,203.6	-
AUC (μg·mL^−1^·min)	499.58	10.3	453.00
C_max_ (μg·mL^−1^)	4.16	10.5	4.92
CL (mL·min^−1^)	3771.20	10.3	3940.05
V_ss_ (mL)	68,464.40	37.4	-
V_d1_ (mL)	68,019.62	148.8	-
V_d2_ (mL)	444.79	19,137.5	-

k_10_: elimination rate constant; k_12_: transfer rate constant from central compartment to tissue compartment; k_21_: transfer rate constant from tissue compartment to central compartment; AUC: area under the plasma concentration-time curve; C_max_: maximum plasma concentration; CL: clearance; V_ss_: steady state volume of distribution; V_d1_: volume of distribution of central compartment; V_d2_: volume of distribution of tissue compartment.

**Table 4:** 5-FU PK parameters obtained from WinNonlin.

5-FU Parameters	Estimate	CV (%)	Literature Values [29]
k_10_ (min^−1^)	9.17 × 10^−2^	4.9	-
k_12_ (min^−1^)	3.21 × 10^−2^	29.5	-
k_21_ (min^−1^)	1.07 × 10^−1^	28.1	-
AUC (μg·mL^−1^·min)	1058.81	1.6	926.80
C_max_ (μg·mL^−1^)	97.14	5.1	-
CL (mL·min^−1^)	850.01	1.6	1069.20
V_ss_ (mL)	12,056.99	4.9	15,912.00
V_d1_ (mL)	9265.14	5.1	-
V_d2_ (mL)	2791.84	14.2	-

k_10_: elimination rate constant; k_12_: transfer rate constant from central compartment to tissue compartment; k_21_: transfer rate constant from tissue compartment to central compartment; AUC: area under the plasma concentration-time curve; C_max_: maximum plasma concentration; CL: clearance; V_ss_: steady state volume of distribution; V_d1_: volume of distribution of central compartment; V_d2_: volume of distribution of tissue compartment.

**Table 5:** Itraconazole PK parameters obtained from WinNonlin.

Itraconazole Parameters	Estimate	CV (%)	Literature Values [28]
k_10_ (min^−1^)	2.80 × 10^−2^	8.8	2.66 × 10^−2^
k_12_ (min^−1^)	2.38 × 10^−2^	9.4	-
k_21_ (min^−1^)	2.34 × 10^−3^	15.3	-
AUC (μg·mL^−1^·min)	437.73	7.9	449.88
C_max_ (μg·mL^−1^)	3.88	0.6	-
CL (mL·min^−1^)	228.45	7.9	246.67
V_ss_ (mL)	90,922.24	20.9	558,000.00
V_d1_ (mL)	8145.37	2.6	-
V_d2_ (mL)	82,776.88	22.9	-

k_10_: elimination rate constant; k_12_: transfer rate constant from central compartment to tissue compartment; k_21_: transfer rate constant from tissue compartment to central compartment; AUC: area under the plasma concentration-time curve; C_max_: maximum plasma concentration; CL: clearance; V_ss_: steady state volume of distribution; V_d1_: volume of distribution of central compartment; V_d2_: volume of distribution of tissue compartment.

## Data Availability

Not Applicable.
